# Hydrogen sulfide and its potential as a possible therapeutic agent in male reproduction

**DOI:** 10.3389/fendo.2024.1427069

**Published:** 2024-09-11

**Authors:** Zuzana Pilsova, Aneta Pilsova, Natalie Zelenkova, Barbora Klusackova, Eva Chmelikova, Pavla Postlerova, Marketa Sedmikova

**Affiliations:** ^1^ Department of Veterinary Sciences, Faculty of Agrobiology, Food, and Natural Resources, Czech University of Life Sciences Prague, Prague, Czechia; ^2^ Laboratory of Reproductive Biology, Institute of Biotechnology of the Czech Academy of Sciences, BIOCEV, Vestec, Czechia

**Keywords:** hydrogen sulfide, male reproduction, sperm, testes, erectile function, antioxidant, vasorelaxation

## Abstract

Hydrogen sulfide (H_2_S) is an endogenously produced signaling molecule that belongs to the group of gasotransmitters along with nitric oxide (NO) and carbon monoxide (CO). H_2_S plays a pivotal role in male reproductive processes. It is produced in various tissues and cells of the male reproductive system, including testicular tissue, Leydig and Sertoli cells, epididymis, seminal plasma, prostate, penile tissues, and sperm cells. This review aims to summarize the knowledge about the presence and effects of H_2_S in male reproductive tissues and outline possible therapeutic strategies in pathological conditions related to male fertility, e. g. spermatogenetic disorders and erectile dysfunction (ED). For instance, H_2_S supports spermatogenesis by maintaining the integrity of the blood-testicular barrier (BTB), stimulating testosterone production, and providing cytoprotective effects. In spermatozoa, H_2_S modulates sperm motility, promotes sperm maturation, capacitation, and acrosome reaction, and has significant cytoprotective effects. Given its vasorelaxant effects, it supports the erection of penile tissue. These findings suggest the importance and therapeutic potential of H_2_S in male reproduction, paving the way for further research and potential clinical applications.

## Graphical Abstract

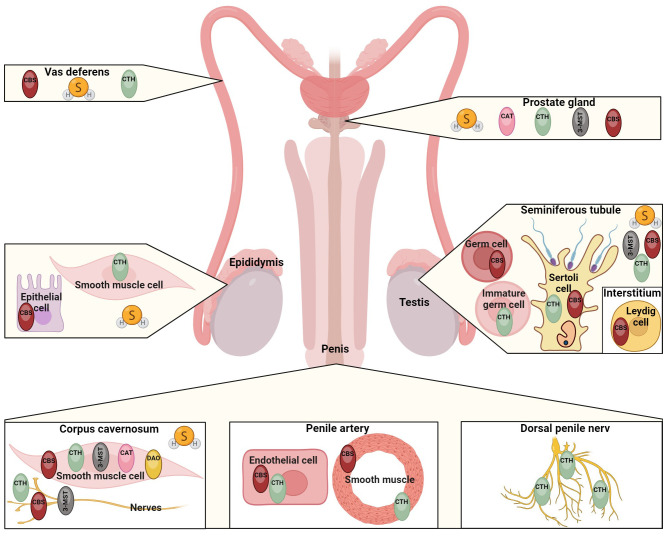

## 1 Introduction

**Figure 1:**
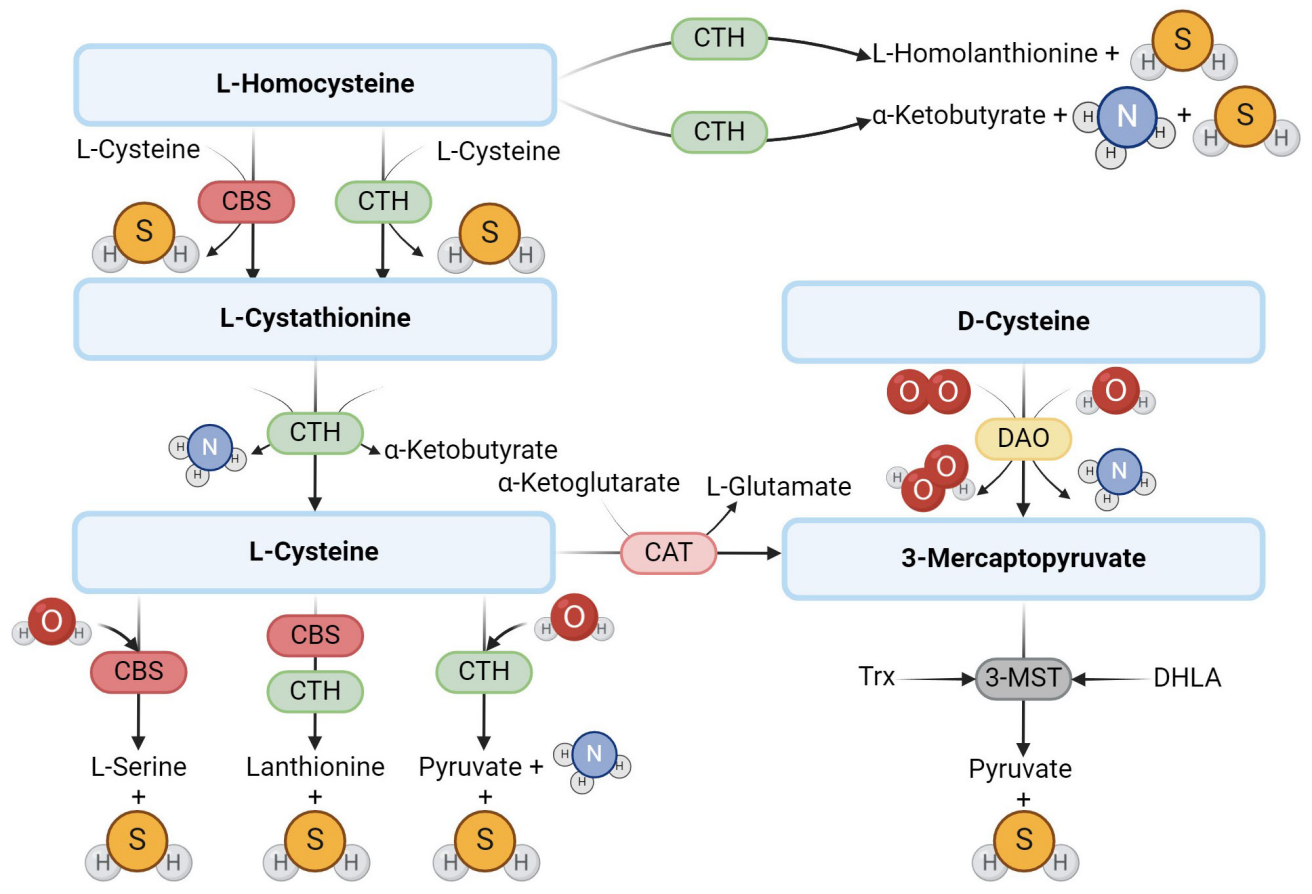
Enzymatic production of endogenous H_2_S. The main producers of H_2_S are the enzymes CBS and CTH, which use L-cysteine to produce H_2_S. Another possible route of H_2_S formation from L-cysteine is the activity of 3-MST and CAT. In addition to L-cysteine, D-cysteine can also be another substrate.

Hydrogen sulfide (H_2_S) is a gasotransmitter, a gaseous signaling molecule that provides cell signaling through a series of intracellular signaling cascades. H_2_S is produced in mammalian tissues via the transsulfuration pathway, which involves the interconversion of cysteine and homocysteine through cystathionine. In mammals, H_2_S is synthesized by the enzymes cystathionine-β-synthase (CBS) and cystathionine-γ-lyase (CTH) (1), which require pyridoxal-5’-phosphate (PLP) as a cofactor and use L-cysteine to produce H_2_S (2). Some studies suggest that the effect of CBS and CTH on L-cysteine is the main pathway to produce endogenous H_2_S (3). However, several other pathways have been described (**Figure 1**). H_2_S can be produced, for example, by 3-mercaptopyruvate sulfurtransferase (3-MST), which is also one of the H_2_S-producing enzymes (1), which, unlike the previous two, is not dependent on PLP. The production of H_2_S through 3-MST also requires cysteine aminotransferase (CAT), which catalyzes the reaction of cysteine with keto acids to form 3-mercaptopyruvate and the corresponding amino acid. 3-MST then catalyzes the conversion of 3-mercaptopyruvate to pyruvate and H_2_S (4, 5) in the presence of reducing agents such as thioredoxin or dihydrolipoic acid (6). In 2013, Shibuya et al. (7) described another pathway of endogenous H_2_S production, which involves two enzymes – diamine oxidase (DAO) and 3-MST. DAO catalyzes the conversion of D-cysteine to 3-mercaptopyruvate, a substrate for 3-MST (7).

The H_2_S production has been described in several mammalian tissues in which H_2_S has a specific function. Endogenous H_2_S uses several signaling pathways to engage in physiological processes. In many mammalian systems, the effect of H_2_S is mediated by ATP-sensitive potassium channels (K_ATP_) (8, 9). Other signaling pathways involve T- and L-type calcium channels (10, 11), large conductance calcium-activated potassium channels (BK_Ca_) (12, 13), MAPK signaling cascade (14), mitochondrial cytochrome c oxidase (15) and transient receptor potential ion channel 1 (TRPA 1) (16). In addition to acting on various signaling pathways, H_2_S is a potent reducing agent that protects sulfhydryl groups of proteins from oxidation thanks to its reactivity with oxygen and nitrogen radicals (17–20). In some tissues, H_2_S deficiency or excess can affect the pathogenesis of some diseases. An insufficient concentration of H_2_S has been described, for example, in Alzheimer’s or Huntington’s disease (17, 21), whereas overexpression of hydrogen sulfide-producing enzymes, such as CTH, is often associated with the presence of testicular neoplasms, embryonic carcinoma (22), or prostate cancer (23, 24). At the same time, this higher expression of CTH correlates with higher aggressiveness of cancer (22, 25, 26). Since early detection is crucial for cancer treatment, new methods have been developed in recent years that can detect H_2_S in these tissues and could also help in the early diagnosis of cancer (27, 28).

**Figure 2:**
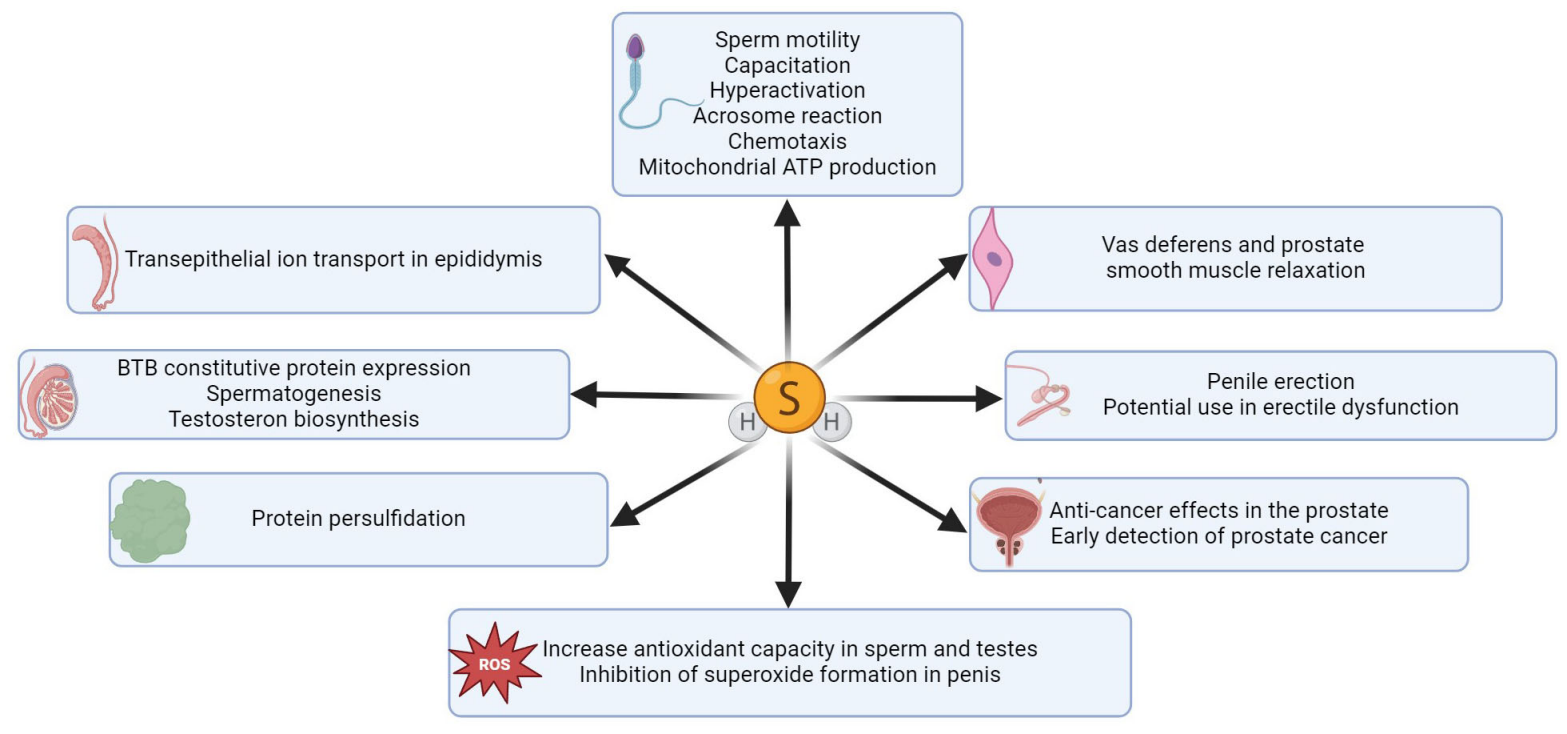
Summary of H_2_S functions in different parts of the male reproductive tract. H_2_S has an impact on various sperm functions, it enhances sperm and testicular antioxidant capacity (38, 49, 50) and inhibits superoxide generation in penile tissue (51, 52). H_2_S effectively promotes erectile function (53), and this gasotransmitter is a promising therapeutic agent for the treatment of erectile dysfunction (42, 54). A key function of H_2_S is protein persulfidation, through which H_2_S can modify proteins and regulate various signaling pathways (55). H_2_S also plays a crucial role in facilitating ion transport across the epididymal epithelium (32) and significantly enhances BTB constitutive protein expression in the testis (31), thereby promoting spermatogenesis and testosterone biosynthesis. H_2_S can also effectively regulate smooth muscle relaxation in vas deferens (33) and prostate tissue (34).

H_2_S and its synthases have also been demonstrated in the male reproductive tract. For example, CBS has been reported in testicular germ cells, Sertoli and Leydig cells, and CTH has been described in immature testicular germ cells and Sertoli cells (29). H_2_S-producing enzymes have also been detected in spermatozoa (30), seminal plasma (31), epididymis (32), vas deferens (13, 33), prostate (24, 34) and penile tissue (35–37). The presence of H_2_S and its synthases in particular parts of the male reproductive tract in different species are listed in **Table 1**. H_2_S is currently considered to be a modulator of physiological sexual function in both sexes (45–48), so it is the reason why many scientific publications have been published in recent years dealing with the function of H_2_S in different parts of the male reproductive tract (**Figure 2**). It has been described that H_2_S increases the antioxidant capacity of sperm (49), has anti-inflammatory and antioxidant effects on testicular cells (50, 56), promotes testosterone production (57, 58) and can support (31, 59) and suppress (31, 32, 60, 61) sperm motility. One of the most critical functions of H_2_S in the male reproductive system is vasorelaxation of smooth muscle, where it helps to relax the smooth muscles of the vas deferens (33), prostate (34) or corpus cavernosum (CC) (35, 53, 62). Smooth muscle relaxation of CC with H_2_S is a highly discussed topic because the treatment of ED in some patients is not sufficient with conventional medications, and H_2_S-based compounds could, therefore, be another possible treatment used for ED patients (42, 54).

**Table 1:** Presence of H_2_S and its synthases in the male reproductive tract.

Localization	Species	Enzyme	Source
Sperm	Human, mouse, boar	CBS, CTH, 3-MST	(30, 31)
Seminal plasma	Human	CBS, CTH	(31)
Testicular tissue	Mouse	CBS, CTH, 3-MST	(30, 38)
Leydig cells	Rat	CBS	(29)
Sertoli cells	Rat	CBS, CTH	(29)
Germ cells of the testicle	Rat	CBS	(29)
Immature testicular germ cells	Rat	CTH	(29)
Epithelial cells of the epididymis	Rat	CBS	(32)
Epididymis smooth muscle cells	Rat	CTH	(32)
Ejaculatory duct	Rat, mouse, human	CBS, CTH	(13, 33)
Prostate	Rat, human	CBS, CTH, 3-MST, CAT	(24, 34)
Corpus cavernosum	Mouse, Human	CTH, CBS	(35, 39–42)
Corpus cavernosum	Mouse	3-MST	(40, 41)
Corpus cavernosum	Rat	CBS, CTH, 3-MST, CAT, DAO	(37)
Penile tissue endothelium	Human, mouse, cattle	CBS, CTH	(39, 43)
Muscular trabeculae of penile tissue	Human	CBS, CTH	(35, 43, 44)
Smooth muscle of the penile artery	Human	CBS, CTH	(35, 43, 44)
Dorsal nerve of the penis	Rat	CTH	(35)

## 2 Hydrogen sulfide in sperm and seminal fluid

**Figure 3:**
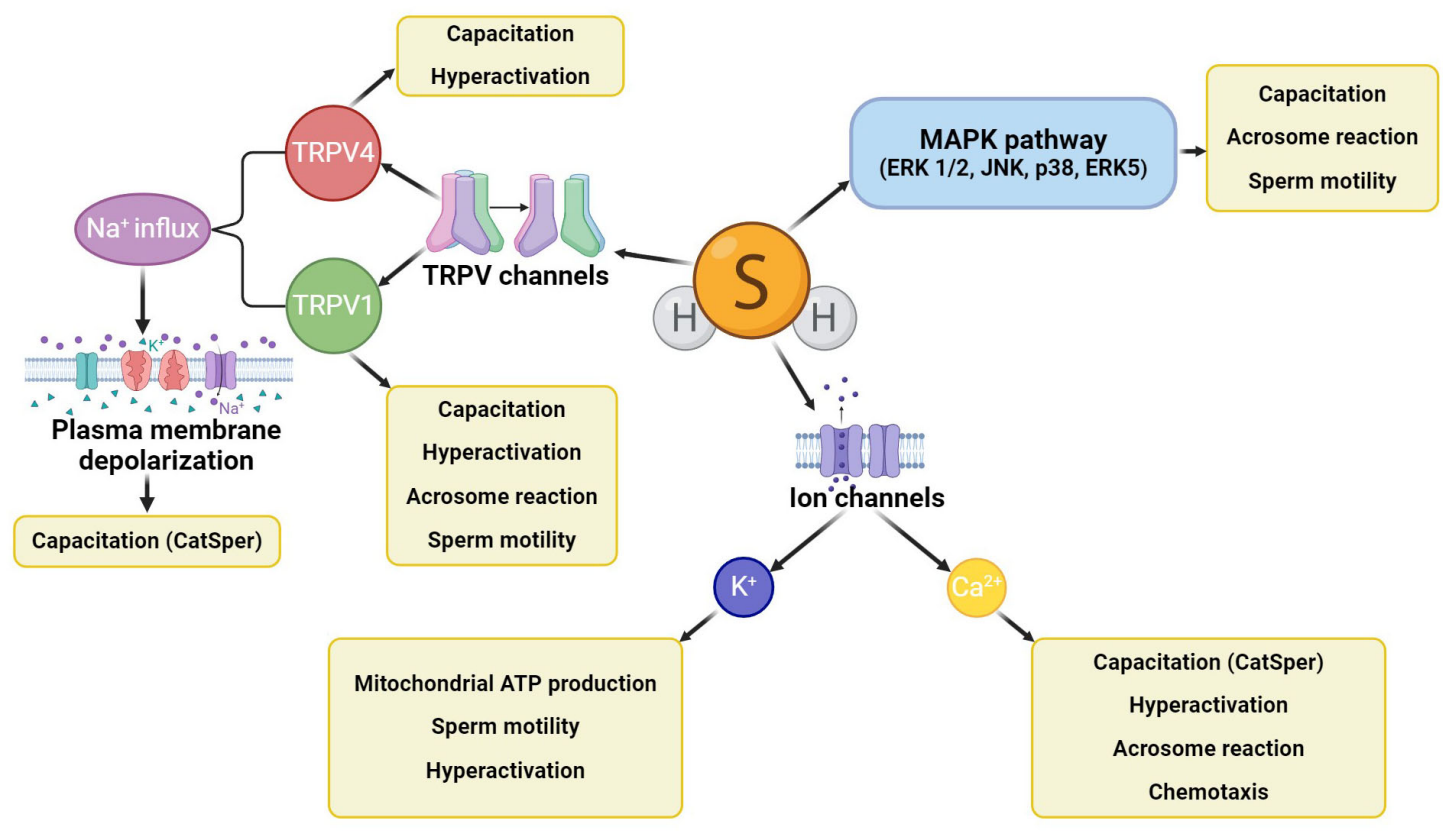
H_2_S signaling cascades in spermatozoa. H_2_S activates the MAPK signaling cascade, further affecting ion channels (mainly potassium and calcium) and some TRPV channels (TRPV1, TRPV4). All these signaling cascades affect sperm motility, capacitation, hyperactivation, or acrosome reaction, and CatSper channels can also engage sperm chemotaxis ability.

H_2_S activates several signaling pathways in spermatozoa (**Figure 3**) which participate in the development of sperm motility, capacitation, and acrosome reaction, such as the activating MAPK pathway involving four central cascades (ERK 1/2, JNK, p38, and ERK5) (31, 63). H_2_S also affects sperm ion channels (Ca^2+^, K^+^, Na^+^), which are also concerned with the physiological processes of spermatozoa (64). An example of the Ca^2+^ channel regulation by H_2_S is the cation channels of sperm (CatSper) engaged in capacitation, hyperactivation, acrosome response, and sperm chemotaxis ability (65). In addition, the opening of K^+^ channels by H_2_S regulates ATP production in the mitochondria, which supports progressive sperm motility and hyperactivation (38, 66). Other molecular targets of H_2_S are some of the subfamilies of transient vanilloid receptor proteins (TRPVs) (64). The TRPV1 channel has been described in the acrosome and flagellum of bull spermatozoa, where it promotes progressive motility, capacitation, including hyperactivity, and acrosome reaction (67). Similarly, the TRPV4 channel has been detected in the flagellum and acrosome of human spermatozoa and is involved in sperm capacitation associated with motility hyperactivation. In addition, TRPV1 mediates Na^+^ influx and subsequent membrane depolarization, activating other important ion channels related to sperm capacitation (e.g., CatSper) (68, 69).

### 2.1 Role of hydrogen sulfide in sperm motility

H_2_S-producing enzymes are mainly found in the midpiece of the human, boar, and mouse sperm flagellum, but during epididymal maturation and capacitation, their sequential disappearance occurs probably due to plasma membrane remodeling (30). These results suggest that H_2_S is not involved in oocyte fertilization alone but in the preceding processes. Given the presence of H_2_S-producing enzymes in the midpiece of the flagellum, it seems likely that H_2_S could be involved primarily in the development of sperm motility by promoting ATP production in mitochondria. The effect of H_2_S on sperm motility is the subject of many research studies describing both the positive and negative effects of H_2_S on this important functional sperm parameter. One of the negative impacts may be the action of the sodium sulfide (Na_2_S) donor, which releases H_2_S rapidly in high concentrations, *in vitro* reducing sperm motility (70). Reduction of sperm motility by H_2_S includes decreasing Na^+^/K^+^ ATPase activity, which is known to affect spermatogenesis, metabolism (71), and sperm motility of various mammalian species (e.g., mice, stallions, humans) (72–75), and protein kinase B (Akt) levels, activating adenosine 5’-monophosphate (AMP)-activated protein kinase (AMPK) and phosphatase and tensin homolog deleted on chromosome ten (PTEN), and increasing reactive oxygen species (ROS) (70). This result has also been confirmed by Wang et al. (61) in an *in vivo* study on mice, which described the same negative effect of a donor-different gasotransmitter (NaHS) on sperm chemotactic abilities when treated at a dose of 50 mg/kg daily. The reason for the negative impact of H_2_S donors on sperm motility is probably the fact that some H_2_S donors release this gasotransmitter in concentrations that are supraphysiological and, therefore, unfavorable for sperm function, which can cause a decrease in sperm motility (70).

Compared to these results, a positive effect of H_2_S donors on sperm motility has also been described in oligoasthenozoospermic (ejaculate with reduced sperm concentration and motility) and asthenospermic (ejaculate with reduced sperm motility) ejaculates, in which lower activity of CBS in seminal plasma was measured in comparison to healthy men. Spermatozoa of these patients were *in vitro* exposed to two H_2_S donors – GYY4137 (10 µM), releasing H_2_S slowly in low concentrations, and NaHS (5 µM), rapidly releasing H_2_S in high concentrations. The first of the mentioned donors (GYY4137) led to improved sperm motility in contrast to NaHS (31). However, improvement in sperm motility occurred only in patients with lower levels of H_2_S (< 18 µM) in seminal plasma, which is probably related to the activation of the MAPK signaling pathway (31), which fundamentally affects sperm motility, morphology, and capacitation (76, 77). Other effective molecules in terms of sperm motility and concentration are the H_2_S precursor SG1002 (*in vivo* study on men, 750 mg of SG1002 daily) (78) and finally, the amino acid-derived N-thiocarboxyanhydrides (NTAs), which releases H_2_S in the presence of carbonic anhydrase, *in vitro* increasing progressive sperm motility and prolonging sperm survival through supported mitochondrial activity (59). These results suggest that H_2_S concentration probably plays a crucial role in the effect on sperm motility.

### 2.2 Hydrogen sulfide as an antioxidant in spermatozoa

One of the leading causes of male infertility is oxidative stress, so experiments have been conducted focusing on using various H_2_S donors, which are considered potent antioxidants (49, 55). The donor GYY4137 releasing H_2_S slowly in small concentrations can maintain sperm motility *in vitro*, even under conditions of increased oxidative stress. Conversely, the *in vitro* effect of NaHS, which releases H_2_S rapidly in high concentrations, preserves sperm motility only in lower concentrations, while in higher concentrations (300 μM), it appears to be cytotoxic. However, both mentioned donors and amino acid NTAs can mitigate damage caused by oxidative stress, thereby increasing sperm antioxidant capacity (49, 59). In addition to its direct ability to neutralize ROS (like O_2_H, O_2_^-^, OH, etc.), H_2_S also enhances the expression of antioxidant enzymes, for example, glutathione peroxidase (GSH-Px) or superoxide dismutase (SOD), through nuclear factor erythroid-derived 2 (Nrf2) activation and translocation, which regulates the expression of antioxidant proteins (79).

### 2.3 Hydrogen sulfide in persulfidation of sperm proteins

**Figure 4:**
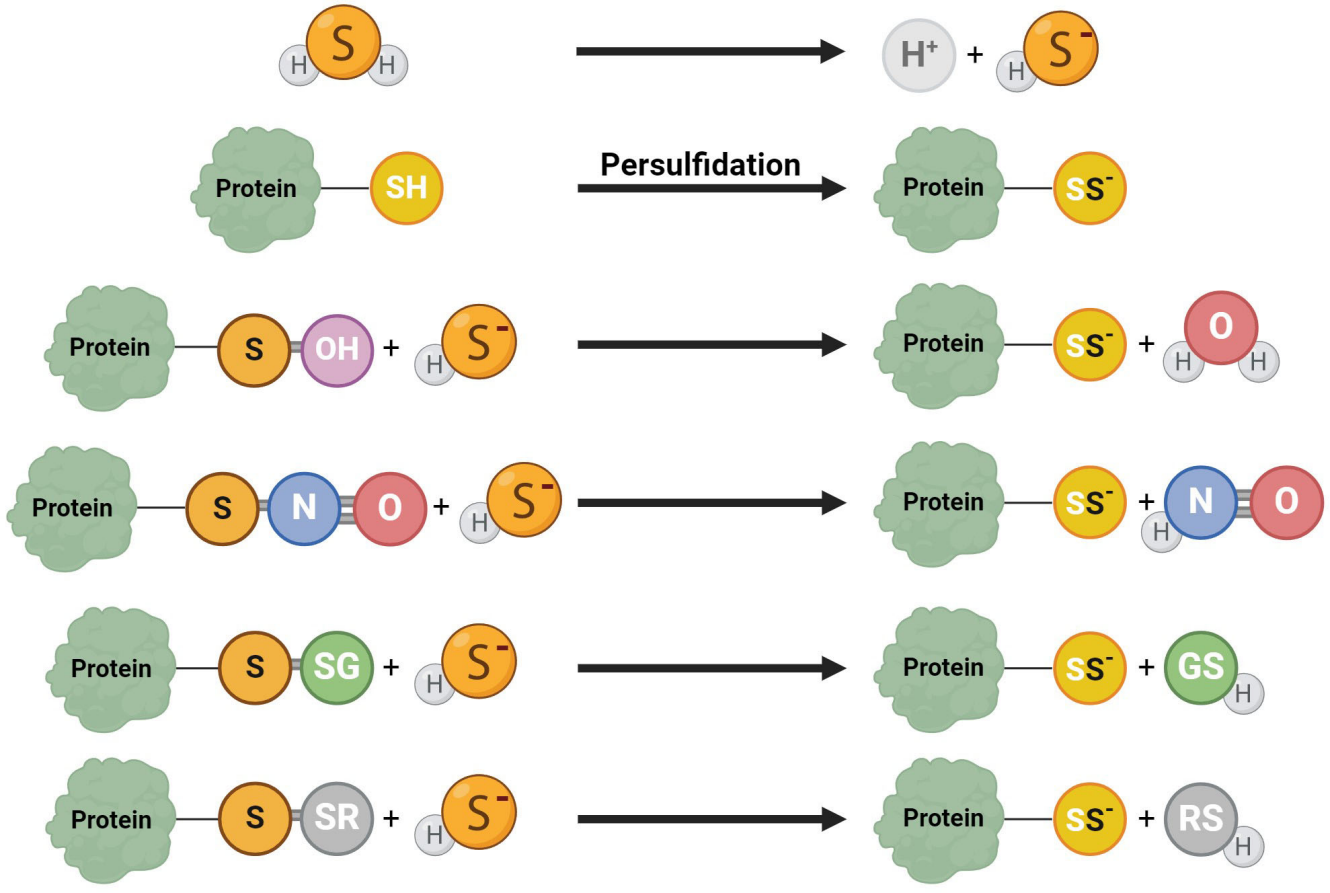
Various reaction mechanisms of persulfidation. In solutions, H_2_S dissociates into H^+^ and HS^−^. Protein persulfidation can result from sulfide anion reactions on oxidized protein thiol, including S-OH, S-N=O, S-SG, and S-SR.

H_2_S facilitates the persulfidation (S-sulfhydratation) (55, 80), posttranslational modification converting thiols (RSH) to persulfides (RSSH, RSSSH, etc.) (**Figure 4**) (81) when H_2_S modifies proteins by attaching a sulfhydryl group to specific cysteine residues (82). This protein modification is a redox process, and it is significant in many signaling pathways relevant to sperm development (48). This modification can be reversible and dynamic, allowing precise regulations of protein functions (83, 84). Persulfidation prevents protein damage caused by irreversible cysteine hyperoxidation, which can be detrimental to protein function (30). Persulfidation protects proteins from irreversible oxidative damage, particularly in environments with high oxidative stress (83, 84). In the context of male reproductive health, this protective mechanism is crucial for maintaining the integrity of sperm proteins, which are vital for sperm motility and function. Persulfidation has been shown to be relatively common in the testes (30), with almost 244 identified persulfidated proteins in human sperm. Interestingly, in asthenozoospermic patients, histones H3.1 and H3.3 on cysteine 111 exhibit significantly lower levels of persulfidation, potentially leading to reduced sperm motility and contributing to infertility (55). Proteins like GADPH, tubulin, and anchor protein A-kinase, which are involved in flagellum structure and sperm motility, are identified as persulfidated (30, 83). The interaction of H_2_S with GADPH, a critical enzyme for sperm motility, enhances cysteine reactivity, indicating that persulfidation could be responsible for the beneficial effects of H_2_S on sperm movement (80).

## 3 Hydrogen sulfide in testes

### 3.1 Hydrogen sulfide function in the blood-testicular barrier

Endogenous production of H_2_S has been confirmed in testicular tissue, and it is assumed that its production is necessary for spermatogenesis (30, 31, 38). Reduced H_2_S production results in impaired spermatogenesis, decreased MAPK phosphorylation, and disruption of the BTB, which plays a critical role in spermatogenesis. Conversely, the presence of H_2_S *in vivo* increases the resistance and expression of the BTB constitutive protein under conditions of increased oxidative stress (31). However, the effect of H_2_S in this tissue is highly dependent on its concentration, as is the case in spermatozoa. On the contrary, *in vivo*, elevated H_2_S levels induced by some donors (NaHS, Na_2_S) can damage BTB integrity, decrease BTB-related gene expression rates, reduce testosterone levels, damage seminiferous tubules, and increase p38 MAPK phosphorylation, a signaling pathway, which regulates cell proliferation, differentiation, apoptosis, and stress response (31). These effects are thought to be due to inhibiting ATP production, specifically mitochondrial Complex IV (85).

### 3.2 Hydrogen sulfide as an antioxidant in testes

Antioxidant, antiapoptotic, and anti-inflammatory effects of H_2_S have been observed in various systems (38, 86–92). Therefore, the H_2_S-producing enzymes CBS and CTH play an essential role in the testis by synthesizing the antioxidant GSH-Px in the transsulfuration pathway, promoting male fertility. Decreased expression of CBS and CTH in testicular tissue leads to a reduced ability to respond to oxidative stress, which may cause a decrease in sperm concentration and motility (56). The antioxidant capacity of H_2_S consists in the activation of SOD, which reduces the level of ROS in testicular germ cells. H_2_S can further promote mitochondrial function, increase ATP production, and decrease ROS production (38, 50). In addition, Mao et al. (93) described a beneficial effect of H_2_S in attenuating acrolein-induced Sertoli cell and germ cell damage *in vitro*, which underlies several reproductive injuries (94–97), suggesting that H_2_S might be used in the future to prevent and treat acrolein-related reproductive injury.

The antioxidant effect of H_2_S is often associated with reducing ischemia-reperfusion injury in treating testicular torsion. By measuring the levels of some antioxidant substances (SOD, reduced glutathione) and oxidative stress, it was concluded that H_2_S contributes to anti-inflammatory, antioxidant, antiapoptotic, and antifibrotic activities in the treatment of testicular torsion (90, 92) via inhibition of inflammatory cytokines (92). H_2_S donor GYY4137 has been confirmed to *in vivo* protect against ischemia-reperfusion injury and attenuate histopathological changes after testicular torsion/detorsion, as well as mediate an increase in antioxidant capacity (89, 92), reduce apoptosis of spermatogenic cells, and increase the expression level of heat shock protein 70 (89), which helps prevent cell apoptosis during heat stress in testicular cells (98), preserve sperm motility in cryopreserved bull sperm (99), and protect proteins and DNA under stress conditions (100). According to the results of these studies, H_2_S treatment has beneficial effects on biochemical and histopathological damage in testicular torsion. Another study focusing on the protective function of H_2_S investigated the *in vivo* effect of H_2_S on the heat stress of testicular germ cells. Heat exposure significantly reduced endogenous H_2_S production and CBS and CTH expression in testes. NaHS application (5,6 mg/kg) alleviated heat stress in testicular germ cells and induced cell death and apoptosis. The number of apoptotic cells was significantly lower, suggesting that H_2_S may protect testicular germ cells through its anti-apoptotic effects (38), likely mediated by the inactivation of the intrinsic apoptosis pathway, as the Bax/Bcl-2 protein expression ratio was reduced but caspase activity was unchanged (38, 89). NaHS also improved mitochondrial function by reducing oxygen consumption and increasing ATP production, SOD activity was stimulated, and ROS production was reduced. Consequently, exogenous H_2_S may protect germ cells by preserving mitochondrial function and stimulating antioxidant activity (38).

The protective effect of H_2_S has also been studied *in vivo* concerning varicocele. It has been confirmed that the use of GYY4137 (5–20 mg/kg) alleviates damage to the testis and ductus epididymis and reduces the number of apoptotic epithelial cells in the epididymis, likely due to a reduction in the levels of caspase-3 and Bax (88, 101). GYY4137 also reduces oxidative stress markers and increases this tissue’s antioxidant activity. In addition, it likely activates the phosphatidylinositol 3-kinase/protein kinase B (PI3K/Akt) pathway, which regulates the cell cycle and is associated with increased sperm motility and may counteract the effects of oxidative stress (101).

Due to the protective effects of H_2_S, its effects have also been investigated about testicular toxicity and male infertility induced by anticancer drugs. Several studies focusing on this topic have described a positive effect of H_2_S against testicular toxicity. As an example, the *in vivo* experiment by Azarbarz et al. (102) confirmed that NaHS administration (200 µg/kg/day) provides a significant improvement in biochemical, histological, and morphometric changes (decrease in testicular weight, plasma testosterone concentration, seminiferous tubule diameter, germinal epithelium thickness, Sertoli cell count, spermatogonia and spermatocytes, Johnsen testicular score and testicular antioxidant enzymes induced by cisplatin (102). Cisplatin is a potent anticancer drug, but its use is limited due to its ability to generate free radicals that are highly toxic to specific organs, such as the kidney and testis (103). A similar *in vivo* experiment was performed by Özatik et al. (91), who investigated the effect of NaHS on testicular dysfunction induced by cyclophosphamide, a drug used to treat many malignancies but which can also cause serious side effects such as hemorrhagic cystitis and male infertility. NaHS (25–100 µmol/kg) has been found to prevent the increase in interleukin 6 and 10 levels, decrease in cGMP, increase in luteinizing hormone (LH), and decrease in testosterone that has been related to the effects of cyclophosphamide (91). Testicular toxicity can also be caused by the carcinogenic water-soluble acrylamide, which is used in paper and plastics manufacturing (104) and has been detected in some cosmetic products, creams, and lotions (105). In addition, acrylamide is also formed naturally during frying or baking at temperatures above 120°C and low humidity (106). Mokhlis et al. (107) described the reversal of the adverse effects of acrylamide-induced testicular toxicity using NaHS *in vivo* and described the protective effects of H_2_S in this exposure (200 µg/kg NaHS) (107). Testicular toxicity can be further induced by nanoplasts, which accumulate in the testes, cause seminiferous tubular degeneration, and induce ROS-dependent mitochondrial apoptosis. Therefore, Li et al. (50) conducted *in vitro* experiments that confirmed the protective function of H_2_S in testicular toxicity. NaHS (250 µM) improved the antioxidant capacity by increasing the protein levels of NAD(P)H dehydrogenase quinone 1 (NQO1) and heme oxygenase-1 (HO-1), which synthesizes CO, another gasotransmitter involved in the regulation of various physiological processes in the body (108). These results suggest that H_2_S donors may be a promising therapy not only for treating varicocele and its symptoms but also for mitigating the adverse effects of anticancer drugs and other agents that cause testicular toxicity.

### 3.3 Hydrogen sulfide and testosterone biosynthesis

**Figure 5:**
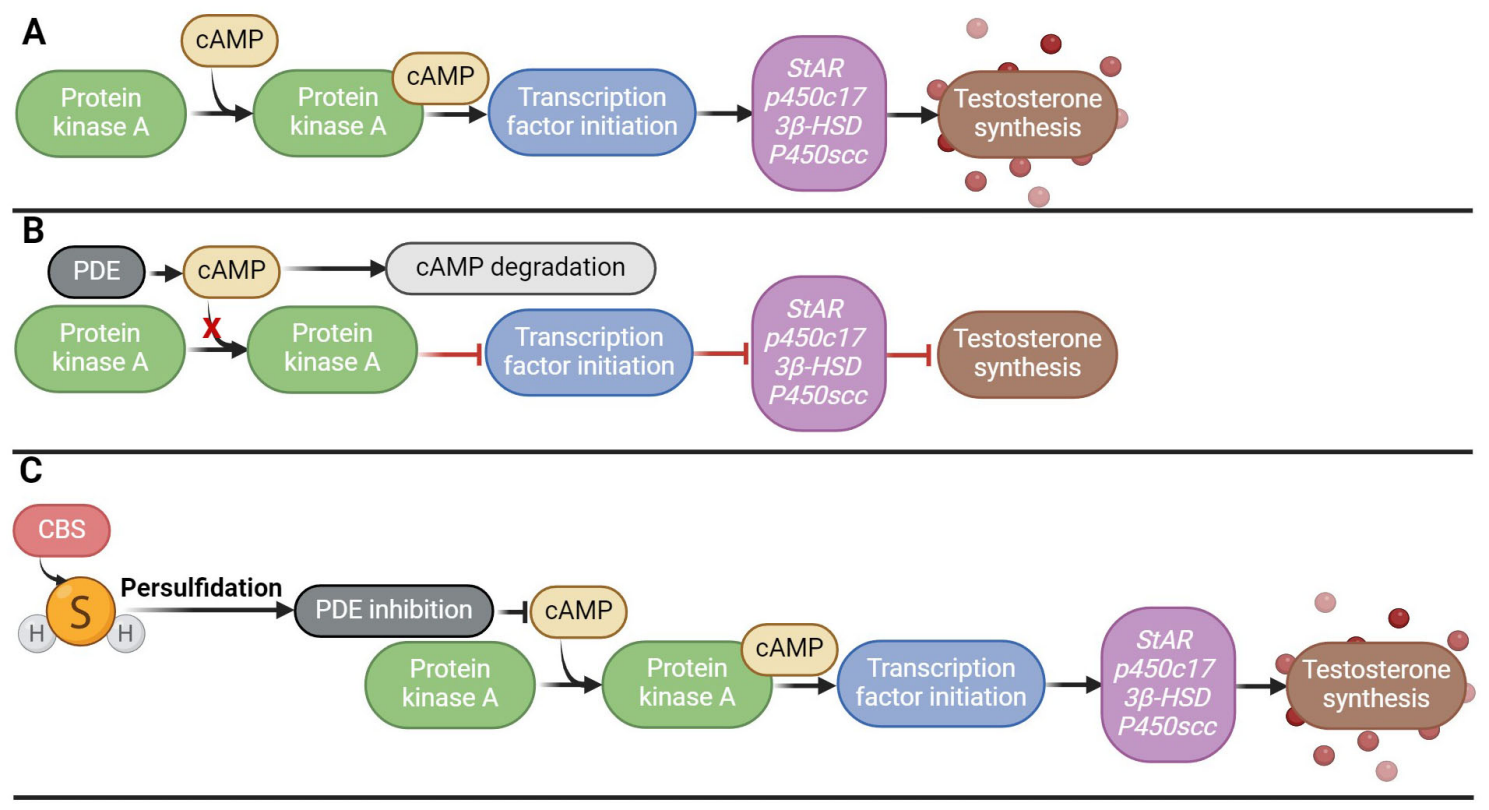
Effect of H_2_S on testosterone biosynthesis. **(A)** Signaling cascade of testosterone secretion; **(B)** Disruption of the signaling cascade of testosterone secretion by overproduction of PDE, which degrades cAMP, which leads to a decrease in the activity of the PKA signaling pathway; **(C)** Persulfidation of PDE by H_2_S and its subsequent inhibition, which ensures activation of the PKA/cAMP signaling pathway and subsequent testosterone synthesis.

Low testosterone levels and hypogonadism occur in 2.1–5.7% of men aged 40–79 years and may be a cause of male infertility (109). However, the crucial role of H_2_S in testosterone biosynthesis has recently been described, the clarification of which could help in the treatment of these disorders associated with low levels of testosterone (58, 61). H_2_S has been described to increase steroid production in Leydig cells and the expression of genes associated with testicular testosterone biosynthesis (*StAR, p450c17, 3beta-HSD, P450scc*) *in vivo*. H_2_S further enhanced SOD and GSH-Px activity, again pointing to its antioxidant effects (58). Another research team looked at the potential solutions to testosterone secretion disorders using H_2_S *in vivo* and *in vitro* (110). Overexpression of CBS was found to inhibit phosphodiesterase 4A (PDE4A) and phosphodiesterase 8A (PDE8A) via persulfidation, implicating that it is possible that by inhibiting PDE expression via persulfidation and activating the cAMP/PKA pathway that regulates testosterone synthesis, which could be restored (**Figure 5**). Furthermore, H_2_S may play an important role in testicular testosterone secretion *in vivo*, which is influenced by LH secretion, because the sulfides contained in garlic support the secretion of testosterone in the testicles precisely by controlling the secretion of LH (111).

## 4 Hydrogen sulfide in epididymis, vas deferens and prostate gland

The role of H_2_S in epididymis is likely to be in regulating epididymal transepithelial ion transport. In rats, H_2_S has been found to induce transepithelial K^+^ secretion via K_ATP_ and BK_Ca_ channels *in vivo*. The activation of BK_Ca_ channels by H_2_S is mediated via TRPV4 channels and subsequent Ca^2+^ influx (32). The expression of the H_2_S-producing enzymes CBS and CTH increases in the epididymis from the caput to the cauda epididymis, corresponding to the increasing production of endogenous H_2_S (32). Moreover, the K^+^ concentration in rat intraluminal fluid also increases from the caput to the cauda, which supports the idea that H_2_S increases K^+^ secretion (32, 112). In the caput epididymis, the function of H_2_S is probably in reducing sperm motility by increasing the extracellular concentration of K^+^ ions, keeping them in a quiescent state before ejaculation (32).

H_2_S production has also been described in vas deferens (13, 33), and all three H_2_S-producing enzymes have been detected in the prostate, with the highest abundance of CTH, which is likely the main H_2_S producer in this tissue (23, 24, 113, 114). The main role of H_2_S in the vas deferens and prostate is primarily to relax smooth muscle (33, 34). In addition, H_2_S also plays an important role in the detection and treatment of prostate cancer. Prostate cancer is the second most frequent malignancy in men and the fifth leading cause of death worldwide (115). As early as 40 years ago, clinical practice demonstrated that water treatment with H_2_S improves prostate blood flow in patients with chronic prostatitis, indicating a beneficial role of H_2_S in prostate tissue in the pathological state (116). Endogenous H_2_S plays a vital role in tumor growth in a variety of cancers through induction of angiogenesis, regulation of mitochondrial bioenergetics, cell cycle acceleration, and antiapoptosis (117), and regulation of cell proliferation (23). *In vivo* and *in vitro* studies have described that H_2_S, its donors, or some sulfocompounds have anticancer effects (23, 118–125). The results suggest that a diet or drug containing H_2_S-releasing substances could be beneficial in the treatment of prostate cancer (23). However, some authors have described that excessive levels of CTH/H_2_S in the prostate, on the contrary, may promote prostate cancer progression and that their inhibition leads to suppression of tumor growth. This suggests that CTH and H_2_S could be potential therapeutic targets in intervening in prostate cancer progression (26). Moreover, the detection of elevated CTH/H_2_S levels could help early prostate cancer detection (27, 28).

## 5 Hydrogen sulfide in penile tissue

The first effect of H_2_S on penile physiology was described in 2006 when intracavernous injection of NaHS (1–10µmol/kg) significantly increased penile length and cavernous pressure in primates (53). A key event during penile erection is the relaxation of the CC smooth muscle, leading to an increase in arterial flow and restriction of venous outflow, thereby resulting in an erection (126–129). Later both *in vitro* and *in vivo* studies suggest that H_2_S functions here as a vasodilator mediator, thereby contributing to blood pressure regulation (39, 130–136). H_2_S is produced in this tissue by both vascular smooth muscle and endothelial cells, on which it has vasodilatory effects (137). Vasodilation is mediated by K_ATP_ (138–142), BK_Ca_ channels (13, 35, 142), TRPVA1 channels, and through the beta3 adrenoceptor signaling pathway (44, 143). The vasodilatory effect of H_2_S is probably enhanced by testosterone, which promotes the production of H_2_S from its precursor, L-cysteine (144), and induces CC smooth muscle relaxation via K_ATP_ channels (145). H_2_S vasodilatory effect has also been demonstrated *in vitro* using its precursor (L-cysteine), donors (GYY4137), and inhibitors (DL-propargylglycine, aminooxyacetic acid) (53, 146, 147). Inhibition of CTH and CBS leads to contraction of the CC and a reduction in the normal intracavernous pressure response to electrical stimulation (35, 43, 53, 148, 149).

**Figure 6:**
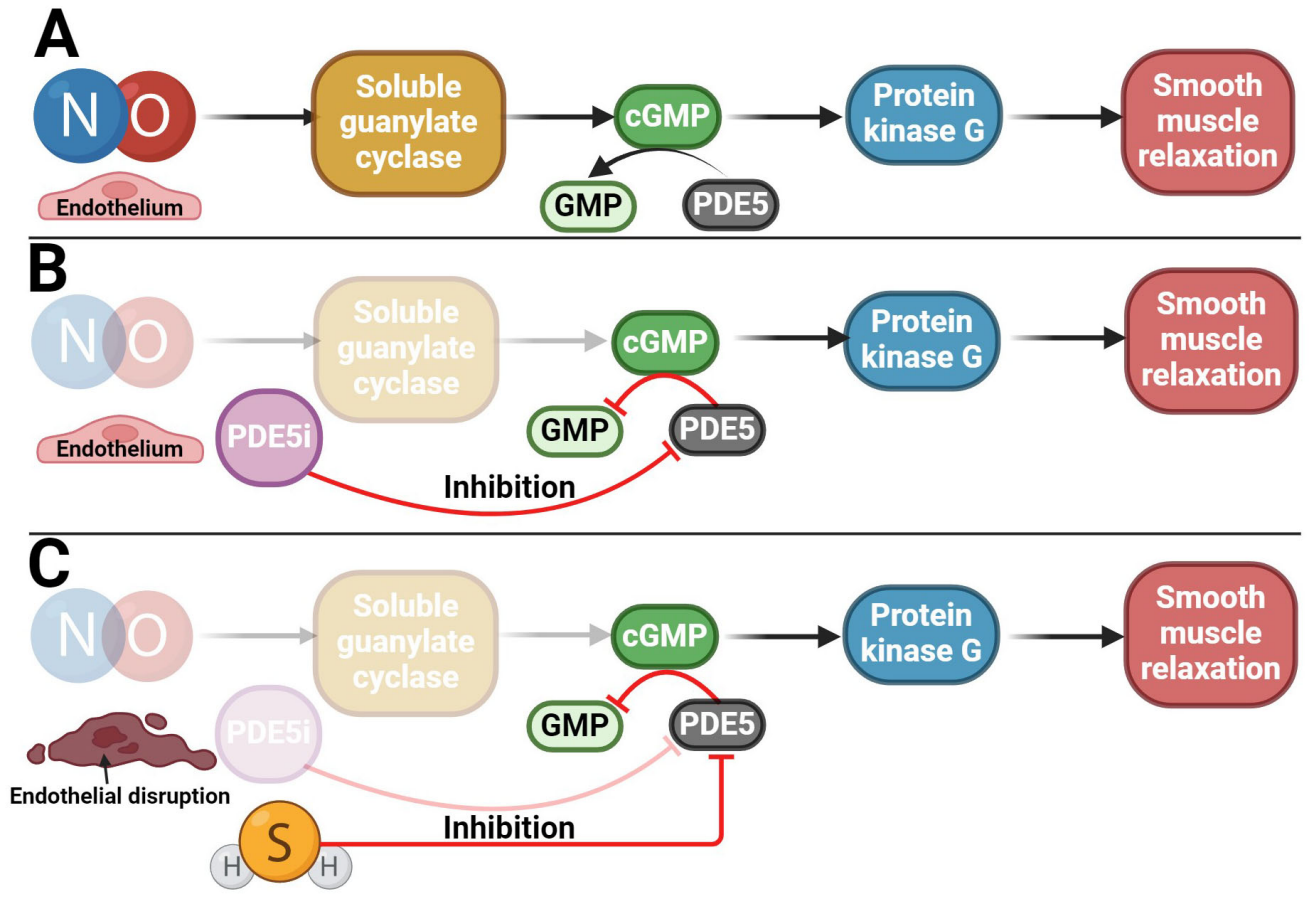
Potential use of H_2_S in the treatment of ED. **(A)** NO/cGMP signaling pathway involved in penile erection; **(B)** Reduction of NO/cGMP signaling pathway activity in patients with ED in whom PDE5i is used as a therapeutic agent, which subsequently preserves the functionality of the signaling pathway; **(C)** Potential use of H_2_S in patients with ED who, due to disruption of the endothelium and the NO/cGMP signaling pathway, do not respond adequately to PDE5i treatment.

In addition, another gasotransmitter contributes to the erection response. A key mechanism of erection lies in the NO signaling pathway, which stimulates the guanylyl cyclase/cyclic guanosine monophosphate (GC/cGMP) pathway (**Figure 6**) (128, 150, 151). H_2_S likely exerts its pro-erectile effects precisely by enhancing the NO signaling pathway (70, 152–155). Indeed, endogenous H_2_S production has been described to significantly enhance the vasorelaxant effect of the NO donor sodium nitroprusside (13, 138, 153, 156–160), and conversely, the H_2_S donor NaHS upregulates endothelial NO synthase (eNOS), thereby relaxing and promoting erectile function (161). Conversely, a decrease in H_2_S levels causes dysregulation of the NO/sGC/cGMP signaling pathway, leading to ED. However, this dysregulation can be reversed by H_2_S donors (162). The pro-erectile effect of H_2_S and its donors likely involves the inhibition of PDEs, resulting in the accumulation of cGMP (163–166). Since PDE proteins contain many cysteine residues, H_2_S is thought to inhibit PDE activity by modifying sulfhydryl sulfides (164). H_2_S alone can enhance cGMP signaling like a PDE5 inhibitor (PDE5i) (**Figure 6**) (164). In addition, H_2_S increased eNOS phosphorylation and xanthine oxidase activity, leading to NO production and increasing NO bioavailability, hence increasing cGMP concentration (166–170). Conversely, NO can modulate endogenous H_2_S production (171). Indeed, it has been found that NO can not only increase the expression of CTH at the transcriptional level but also increase the activity of this enzyme itself, for example, by NO stimulating the uptake of cysteine, which is a substrate for CTH (172). The role of both gasotransmitters in regulating erectile function is likely to be synergistic (153, 155, 173). Taken together, H_2_S acts in CC by at least two mechanisms: inhibiting PDE and activating K_ATP_ channels modulated by testosterone. However, what can be inferred from the available data is that there is an interplay between H_2_S and NO/cGMP in CC and that the on/off switching of signaling may be reciprocally regulated and influenced by testosterone (174). These data imply a strong link between aging, testosterone, H_2_S, and ED (164).

The occurrence of ED is most often related to a disorder of the blood supply to the penile arteries. The development of ED usually occurs during aging (148) or due to other diseases such as diabetes (37, 175), hypertension (175, 176), or metabolic syndrome (177). PDE5i are widely used to treat ED (178) due to its capability to increase cGMP and/or cAMP levels, leading to activation of PKG/PKA and smooth muscle relaxation in erectile tissues (179–181). Some patients, though, specifically elderly, diabetics, and hypertensives, respond inadequately or not at all to PDE5i (182–184). In addition, they often have impaired H_2_S and testosterone signaling (37, 42, 148, 185, 186). In patients who do not respond to PDE5i therapy, a possible clinical treatment for ED could be the use of a combination of PDE5i, testosterone, and/or H_2_S donors/substrates, which could lead to cGMP levels maintenance (35, 40, 43, 154, 157, 177, 186, 187). The advantage of H_2_S-induced CC relaxation is that it is independent of the endothelium (157, 188, 189), which NO/cGMP pathway is not, and it is one of the reasons why, for example, diabetic patients develop ED (**Figure 6**) (190–193). As a result, it can be said that H_2_S has a compensatory role in the absence of NO without altering the downstream mechanisms of the signaling pathway. However, the potential therapeutic use of H_2_S could lie not only in its acute effect on erection but also in its longer-term effect in reducing oxidative stress in erectile tissue, both of which have been described in another H_2_S donor – a sildenafil derivative ACS6 (52, 157, 194–196).

## 6 Conclusion

Endogenous production of H_2_S, which plays a crucial role in the processes of male reproduction, has been confirmed in most tissues of the male reproductive system. H_2_S is essential in the early phase of male fertility, as it maintains the integrity of BTB during spermatogenesis, promotes testosterone production, and has cytoprotective effects. Cytoprotective effects may be used, for example, in the treatment of testicular cancer as a means of mitigating the impact of testicular toxicity induced by anticancer drugs. The vasodilatory effects of H_2_S and the subsequent improvement in tissue blood flow could support the treatment of chronic prostatitis. Cytoprotective effects may, in turn, help in the treatment of prostate cancer. At the same time, H_2_S could also aid in the early detection of prostate cancer as a marker for which supraphysiological levels of H_2_S are typical.

In spermatozoa, H_2_S both inhibits sperm motility during epididymal maturation and supports its hyperactivation during capacitation. H_2_S is also involved in the acrosome response of sperm and protects sperm from oxidative stress throughout the reproductive maturation process. These effects are mediated primarily by the action of H_2_S on mitochondria, ion channels (Ca^2+^, K^+^, TRPV), the MAPK signaling pathway, or the persulfidation of sperm proteins. The effect of H_2_S on sperm motility could be exploited in treating patients with reduced sperm motility, for example, by providing an exogenous H_2_S donor. The physiological concentration of H_2_S in the body is relatively low, so donors would be needed for possible treatment that releases H_2_S in low concentrations, such as GYY4137, SG1002, or NTAs.

The most researched area of this topic is the penile tissue, as H_2_S can cause or promote pro-erectile effects. The latter through its relaxant effects on the CC, mediated by K_ATP_ channels, or by inhibiting PDE or upregulating eNOS, leading to relaxation of CC mediated by the NO/GC/cGMP signaling pathway. The synergy of H_2_S and NO in penile tissue works both ways, promoting CTH activity through NO. Due to its pro-erectile effects, H_2_S is a potential therapeutic agent for treating ED patients who do not respond to PDE5i therapy or have impaired endothelial NO production in the CC, such as diabetic patients.

The H_2_S concentration is a crucial factor in its tissue effects. In previous studies, two main types of H_2_S donors have been investigated. Namely, some donors release H_2_S rapidly and in high concentrations (e.g., NaHS) or release H_2_S slowly in low concentrations (GYY4137). While the second group of donors better mimics the physiological concentrations, it is worthwhile for future research to investigate the physiological effects of H_2_S using these donors. The first group of donors, such as NaHS, forming supraphysiological levels of H_2_S in cells may cause tissue or cellular toxicity or promote cancer.

In conclusion, H_2_S may be a valuable tool in the treatment of many pathological conditions of male reproduction, such as impaired spermatogenesis, lack of sperm motility, or ED. However, for the successful application of H_2_S in clinical settings, it is important to determine the precise concentrations that elicit the desired therapeutic effects. Furthermore, future research will likely uncover specific protein targets of persulfidation and elucidate how this posttranslational modification influences their function. This understanding will be crucial in identifying and defining critical therapeutic targets, ultimately paving the way for more targeted and effective treatments.
